# An Insight into Recombination with Enterovirus Species C and Nucleotide G-480 Reversion from the Viewpoint of Neurovirulence of Vaccine-Derived Polioviruses

**DOI:** 10.1038/srep17291

**Published:** 2015-11-25

**Authors:** Yong Zhang, Dongmei Yan, Shuangli Zhu, Yorihiro Nishimura, Xufang Ye, Dongyan Wang, Jaume Jorba, Hui Zhu, Hongqiu An, Hiroyuki Shimizu, Olen Kew, Wenbo Xu

**Affiliations:** 1 WHO WPRO Regional Polio Reference Laboratory and Ministry of Health Key Laboratory for Medical Virology, National Institute for Viral Disease Control and Prevention, Chinese Center for Disease Control and Prevention, Beijing, People’s Republic of China; 2 Department of Virology II, National Institute of Infectious Diseases, Tokyo 208-0011, Japan; 3 Guizhou Center for Disease Control and Prevention, Guiyang 550004, People’s Republic of China; 4 Division of Viral Diseases, National Center for Immunization and Respiratory Diseases, Centers for Disease Control and Prevention, Atlanta, Georgia 30333, USA

## Abstract

A poliomyelitis outbreak caused by type 1 circulating vaccine-derived polioviruses (cVDPVs) was identified in China in 2004. Six independent cVDPVs (eight isolates) could be grouped into a single cluster with pathways of divergence different from a single cVDPV progenitor, which circulated and evolved into both a highly neurovirulent lineage and a less neurovirulent lineage. They were as neurovirulent as the wild type 1 Mahoney strain, recombination was absent, and their nucleotide 480-G was identical to that of the Sabin strain. The Guizhou/China cVDPV strains shared 4 amino acid replacements in the NAg sites: 3 located at the BC loop, which may underlie the aberrant results of the ELISA intratypic differentiation (ITD) test. The complete ORF tree diverged into two main branches from a common ancestral infection estimated to have occurred in about mid-September 2003, nine months before the appearance of the VDPV case, which indicated recently evolved VDPV. Further, recombination with species C enteroviruses may indicate the presence and density of these enteroviruses in the population and prolonged virus circulation in the community. The aforementioned cVDPVs has important implications in the global initiative to eradicate polio: high quality surveillance permitted earliest detection and response.

High frequency of genetic changes, including nucleotide substitutions and recombination, occur during the lifecycle of wild polioviruses when they replicate in human guts^1^,^2^,^3^. The live, attenuated oral polio vaccine (OPV), which was successfully used for controlling and preventing the circulation of wild polioviruses in the World Health Organization (WHO) program for the global eradication of poliomyelitis, also frequently undergoes such genetic changes throughout their genomes while replicating in human guts because of their inherent genetic instability^1^,^2^,^4^. The genetic instability of OPV strains due to a RNA-dependent RNA polymerase error and recombination also appear to underlie the occurrence of poliomyelitis outbreaks associated with circulating vaccine-derived polioviruses (cVDPVs), which exhibit ≤99% (for type I and type III) or ≤99.5% (for type II) *VP1* sequence homology to the OPV strains^5^,^6^. Two genetic characteristics—nucleotide mutations and recombination—seem to underlie the occurrence of poliomyelitis outbreaks associated with cVDPVs^6^,^7^,^8^,^9^.

To date, there have been several outbreaks of cVDPVs worldwide, for example, in Egypt^10^, Hispaniola (Haiti and the Dominican Republic)^8^, The Philippines^9^, Madagascar^11^,^12^,^13^, China^14^,^15^,^16^, Indonesia^17^, Cambodia^18^, Nigeria^19^,^20^, and Afghanistan^21^. Some phenotypic properties of cVDPVs resemble those of wild polioviruses rather than those of vaccine-related polioviruses; these include properties such as the capacity for sustained person-to-person transmission, higher neurovirulence, critical attenuating sites either have reverted or have been exchanged out by recombination, “non-vaccine-like” antigenic properties, the ability to replicate at a higher temperature, and the ability to undergo recombination with non-polio enteroviruses (NPEVs) during circulation. Hence, cVDPVs may greatly hinder the polio eradication initiative taken worldwide, especially in “polio-free” countries such as China^6^,^22^.

From May to August 2004, 3 AFP patients and 4 contacts of these patients associated with cVDPVs infection were reported in Zhenfeng county, Qianxinan prefecture, Guizhou Province, China^15^. The first patient, a 1-year-old boy, lived in Jiaoyang village; he had a 0-dose OPV history, and the onset of paralysis was dated to May 22, 2004. The second (index patient) and third patients lived in Yaoshang village; they were boys aged 3 and 1 years, respectively, with a 0-dose OPV history and the onset of paralysis dated to June 13, 2004 and July 11, 2004, respectively^15^. All the 3 patients had residual paralysis 60 days after the onset, and their condition was classified as poliomyelitis by the Guizhou provincial and National polio diagnosis experts group.

Type 1 VDPVs were isolated from the index (second) patient (CHN8184) and the third patient (CHN8229-1, CHN8229-2, and CHN8229-3, three strains isolated from three successive stool samples of the same patient) and a contact of the first case patient (CHN8233c), and type 1 VDPVs were also isolated from three contacts of the index patient who lived in the same village (CHN8225c, CHN8248c, and CHN8264c). So six independent cVDPVs are described in this report and made up of eight isolates of cVDPVs. The virus circulates when the OPV coverage in a local area is relatively lower, and the circulation ceases after a mass immunization with OPV. Most of the genetic and phenotypic properties of the type 1 cVDPVs (hereafter, Guizhou/China cVDPVs) isolated in this outbreak were indistinguishable from those of wild-type polioviruses, while some properties were similar to those of type 1 cVDPVs isolated in Hispaniola and The Philippines^8^,^9^; however, they also showed some clear differences in the genetic and phenotypic characterizations.

## Results

### Initial characterization of poliovirus isolates

Poliovirus isolates were preliminarily characterized by two intratypic differentiations (ITD) methods that can distinguish vaccine strains from non-vaccine strains, a polymerase chain reaction-restriction fragment length polymorphism (PCR-RFLP) analysis that was based on the genetic properties of polioviruses, and an enzyme-linked immunosorbent assay (ELISA) method that was based on the antigenic properties of the polioviruses using highly specific cross-absorbed antisera^23^. The PCR-RFLP ITD method showed Sabin like (SL) genetic properties, but ELISA ITD method showed non-Sabin like (NSL) antigenic reactivities for all eight Guizhou/China cVDPV strains. Sequencing of the *VP1*-coding region showed that all the 8 isolates shared common nucleotide substitutions at 5 positions different from the Sabin 1 strain. Their sequences differed from those of the Sabin 1 strain by 9–11 nucleotide substitutions (1.0–1.2% difference) in the *VP1*-coding region.

### Emergence of two cVDPV lineages

**Figure 1:**
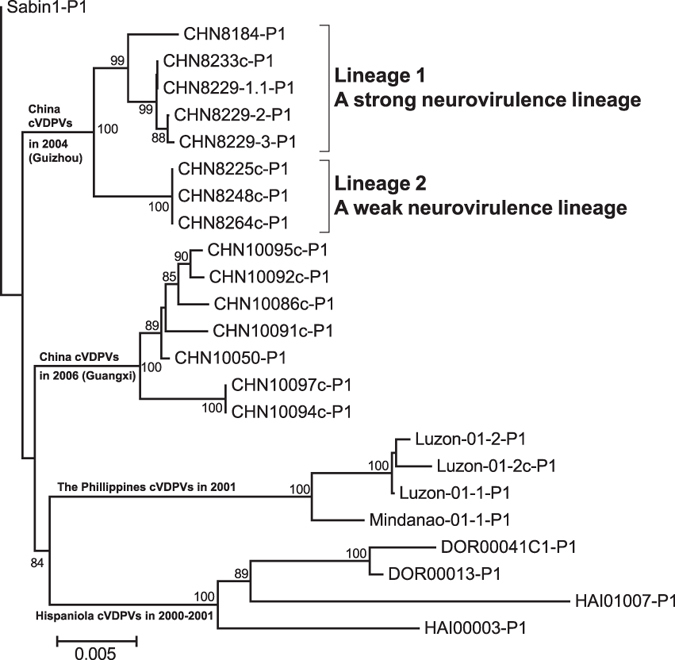
The neighbor-joining tree showing the phylogenetic relationship based on the *VP1*-coding region between the Guizhou cVDPV isolates and other type 1 VDPV strains described previously. The accession numbers are as follows: Guizhou cVDPVs (Genbank accession numbers: FJ769378–FJ769385); Guangxi cVDPVs (Genbank accession numbers: FJ859058–FJ859064); Hispaniola cVDPVs (GenBank accession numbers: AF405666, AF405669, AF405682, and AF405690); The Philippines cVDPVs (GenBank accession numbers: AB180070–AB180073).

In order to elucidate the divergence and evolution of the type 1 Guizhou/China cVDPVs, the *VP1* sequences of the following viruses were aligned and phylogenetic analysis was performed: all the 8 type 1 cVDPVs in this study, Guangxi type 1 cVDPV strains isolated in China in 2006^14^ (GenBank accession numbers: FJ859058–FJ859064), Hispaniola type 1 cVDPVs^8^ (GenBank accession numbers: AF405666, AF405669, AF405682, and AF405690) and the Philippines type 1 cVDPVs (GenBank accession numbers: AB180070–AB180073)^9^ (Fig. 1).

The phylogenetic tree, which was based on the *VP1*-coding region, revealed that all the 8 Guizhou/China type 1 cVDPVs could be grouped into a single cluster with pathways of divergence different from those of Sabin 1, and they were distinct from the genetic clusters of other VDPVs (Fig. 1). Moreover, the Guizhou/China cVDPVs strains could be divided into 2 lineages separated from the single cluster derived from the same root: 5 strains (CHN8184, CHN8229-1, CHN8229-2, CHN8229-3 and CHN8233c) belonged to Lineage 1, which consisted of viruses that infected patients and caused paralysis, while 3 strains (CHN8225c, CHN8248c, and CHN8264c) isolated from 3 contacts of the patients belonged to Lineage 2 (Fig. 1).

### Genetic characterization of Guizhou/China cVDPV strains

**Figure 2:**
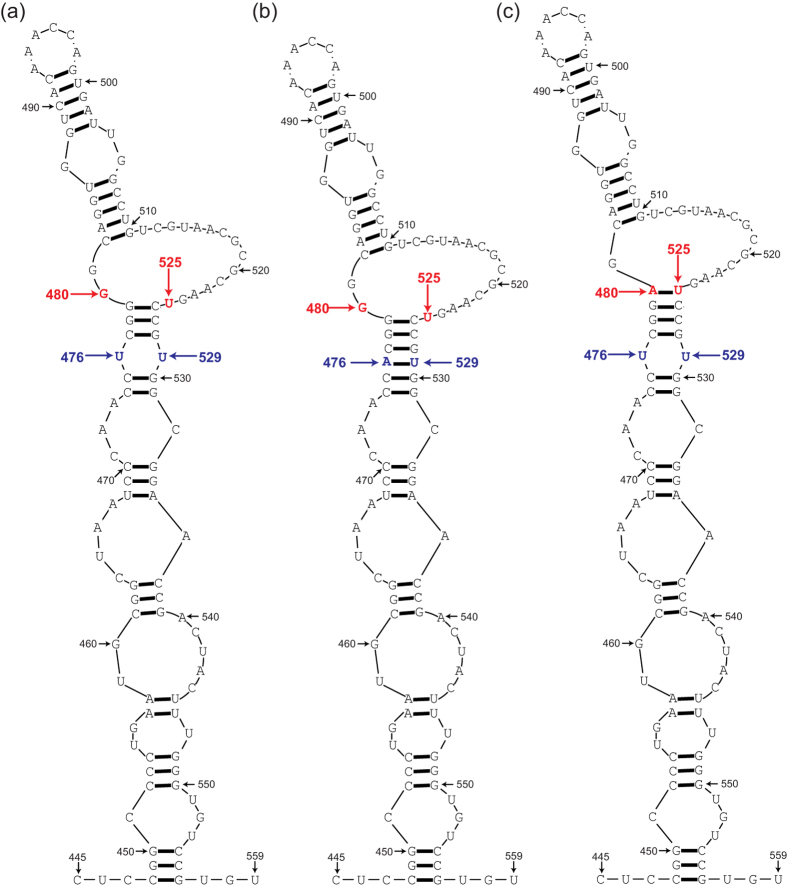
The computer predicted secondary structure of domain V of the internal ribosome entry site (IRES) in the 5′-UTR region of polioviruses. (**a**) Sabin 1 strain; (**b**) Guizhou/China cVDPV strain; (**c**) Mahoney strain. The numerals refer to the positions of the nucleotides of Sabin 1. The attenuated determinants sites 480 and 525 in domain V are highlighted in red and sites 476 and 529 in domain V are highlighted in blue.

The complete genome sequences of 8 Guizhou/China cVDPV strains shared >99.3% nucleotide sequence identities with each other, validating the circulation of cVDPVs in Zhenfeng county. Among the known neurovirulence determinants of type 1 polioviruses^24^,^25^,^26^, the 8 cVDPV strains shared 5 nucleotide reversions: a U-to-A reversion at nt476 in the 5′-UTR region, an A-to-U reversion at nt2438 in the *VP3* region (leading to a Met-to-Leu amino acid substitution), an A-to-G transition at nt2795 in the *VP1* region (leading to a Thr-to-Ala amino acid substitution), a C-to-U transition at nt6203 in the *3D* region (leading to a His-to-Tyr amino acid substitution), and a G-to-A transition at nt7441 in the 3′-UTR region. Strains CHN8229 and CHN8233c exhibited an extra U-to-G reversion at nt935 in the *VP4* region (leading to a Ser-to-Ala amino acid substitution). It is noteworthy that no changes occurred in the nucleotide pair nt480:nt525, which result in the strengthening of a base pair in the main stem region of domain V in the internal ribosome entry site (IRES) in the 5′-UTR region known to have an effect in the reversion of the attenuation phenotype of Sabin 1 (Fig. 2); Mutation at either of these two positions results in a change from a weak base pair GU to a stronger base pair AU or GC which serves to restore the stability of the secondary structure of domain V^27^,^28^. (Table 1).

### Non-recombinant structure of the Guizhou/China cVDPV strains

**Figure 3:**
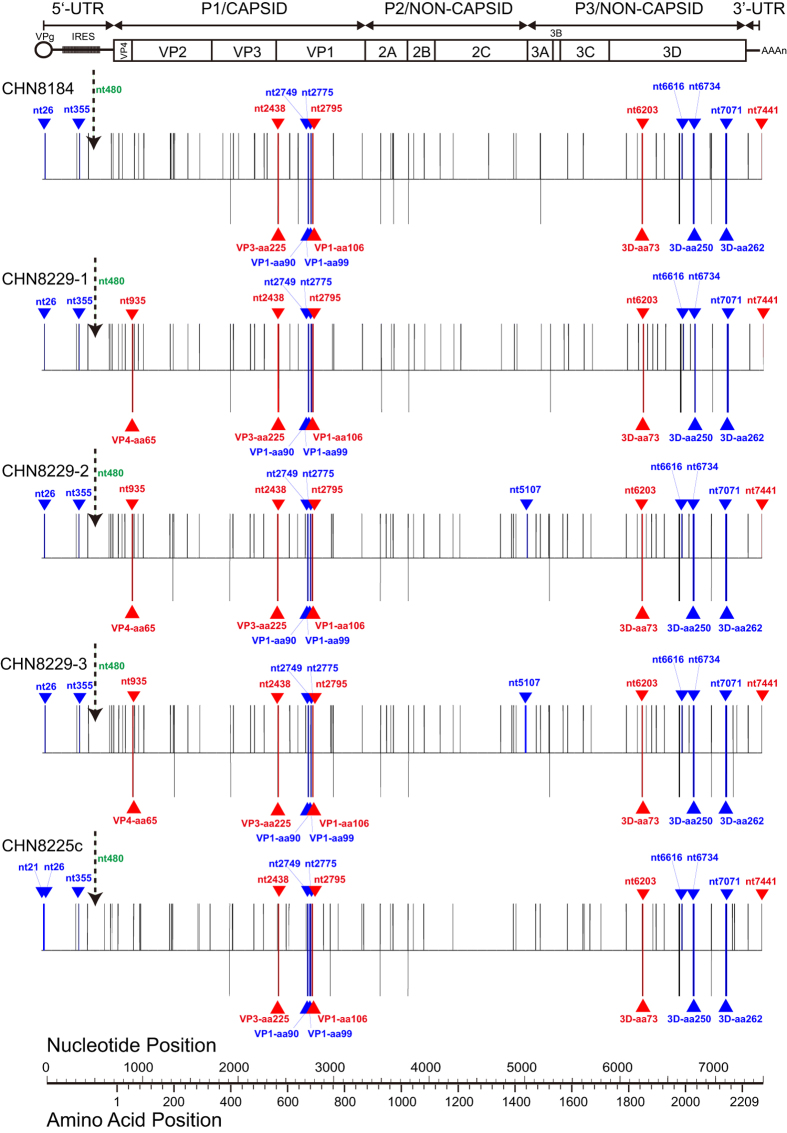
Nucleotide (upper bars) and amino acid (lower bars) substitutions into the genome of China cVDPV isolates. Sabin 1 was used as the reference sequence. The substitution maps are aligned with a schematic of the poliovirus genome. The single open reading frame (ORF), flanked by 5′-UTR and 3′-UTR, is indicated by a rectangle; the internal ribosome entry site (IRES) in the 5′-UTR is indicated by a shaded rectangle. The triangles indicate back mutations to the Mahoney strain, among them, red triangles indicate back mutations related to the general accepted neurovirulence sites and blue triangles indicate back mutations have nothing to do with the general accepted neurovirulence sites; the dashed arrows indicate the absence of back mutation of the major determinant in the IRES (nt480).

All the complete genome sequences of the 8 Guizhou/China cVDPV strains were 7441 nucleotides in length. They showed 65–77 nucleotide substitutions when compared with the reference Sabin 1 strain, and the substitution positions were randomly distributed across the genomic regions (Fig. 3). The complete genome sequence homologies between the Sabin 1 strain and the Guizhou/China cVDPV strains were ≥99.0% in the whole genome sequence and ≥98.9%, ≥99.1%, and ≥98.9% in the *P1*, *P2*, and *P3* capsid region sequences, respectively. These results revealed that all the 8 strains were non-recombinants.

### Changes in neutralizing antigenic sites

**Figure 4:**
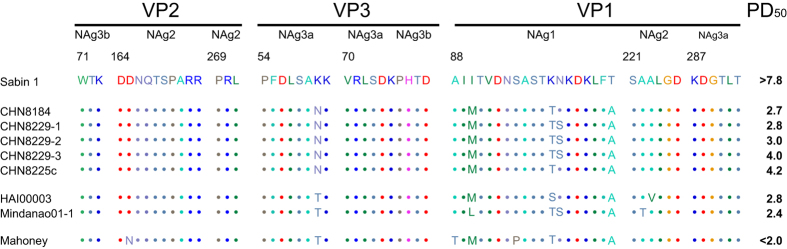
Alignment of amino acid residues of neutralizing antigenic (NAg) sites. NAg sites 1 (*VP1*: 88–106), sites 2 (*VP2*: 164–173; *VP2*: 269–271; *VP1*: 221–226), sites 3a (*VP3*: 54–61; *VP3*: 70–74; *VP1*: 287–292), and sites 3b (*VP2*: 71–73; *VP3*: 75–79) for Sabin 1, Guihzou/China cVDPVs, and Mahoney strains. The PD_50_ value of the representative type 1 cVDPV strains from Hispaniola and The Philippines are cited from the previous reports^8^,^9^.

The ELISA ITD test suggested that the antigenic properties of all the 8 Guizhou/China cVDPV strains differed from those of the reference Sabin 1 strain. Moreover, 13–18 amino acid replacements occurred throughout the capsid region and the noncapsid region of the 8 cVDPV strains, and 7 amino acid reversions to the Mahoney strain were shared among the 8 strains (Fig. 3). The amino acid sequences within or near the predicted neutralizing antigenic (Nag) sites^29^,^30^ of the Sabin 1 strain, its parental Mahoney strain, and representative type 1 cVDPVs from the outbreaks in Hispaniola and the Philippines, were aligned with the five Guizhou/China cVDPV strains (Fig. 4). The five cVDPV strains shared 4 amino acid replacements in the NAg sites: 3 located at the BC loop, which formed the NAg site 1 (VP1–90: Ile-to-Met; VP1–99: Lys-to-Thr; and VP1–106: Thr-to-Ala)^31^, and another at NAg site 3a (VP3–60: Lys-to-Asn). In addition, strains CHN8229-1, CHN8229-2 and CHN8229-3 showed another amino acid replacement at NAg site 1 (VP1–100: Asn-to-Ser) (Fig. 4). These amino acid replacements in the epitopes, especially at NAg site 1, may underlie the aberrant results of the ELISA ITD test. The *Ka*/*Ks* ratio within the NAg sites was 1.66 for the Guizhou cVDPVs, similar to the ratios for type 1 iVDPV isolates from immunodeficient patients in Taiwan (1.07 to 3.04)^32^, and higher than the ratios for the type 1 cVDPVs from Guangxi/China (0.59)^14^, Hispaniola and the Philippines (0.47 to 0.52)^8^,^9^.

### Estimated time of initiating OPV dose

**Figure 5:**
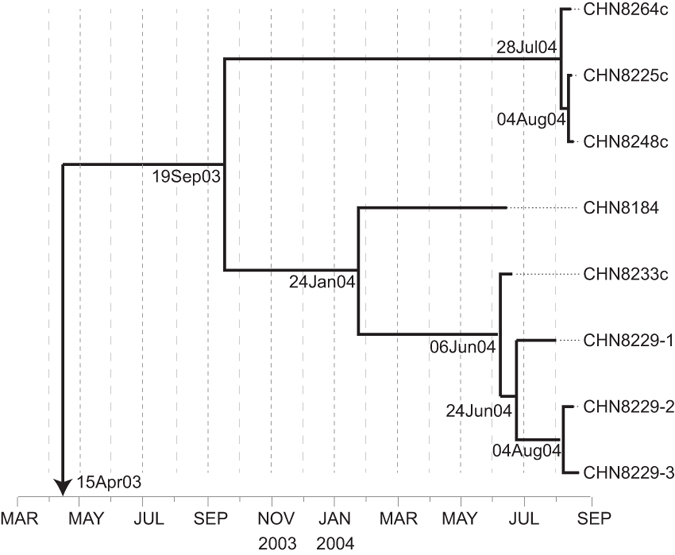
Bayesian Markov chain Monte Carlo tree based on the 3CP of the complete ORF sequences of the 8 Guizhou/China cVDPV isolates rooted to the sequences of Sabin 1 strain. The date of the initiating OPV dose and the times of divergence of different lineages were estimated by assuming a strict molecular clock.

A Bayesian Markov chain Monte Carlo (MCMC) phylogenetic tree was constructed from the sequences at the third-codon position (3CP) of the complete ORF (6,627 nt, nt743 to nt7369) of the eight cVDPV isolates and Sabin 1 strain as a root sequence (Fig. 5). The complete ORF tree diverged into two main branches from a common ancestral infection estimated to have occurred in about mid-September 2003, nine months before the appearance of the VDPV case. Under the assumption of a strict molecular clock with a fixation rate of 3.4 × 10^−2^ 3CP substitutions/site/year (overall rate, 1.2 × 10^−2^ total substitutions/site/year)^33^, we estimated that the initiating OPV dose was given in April 2003, 14 months before the appearance of the first VDPV case (Fig. 5).

### Guizhou/China cVDPV strains appeared high neurovirulence

Strains CHN8184 and CHN8229 were isolated from patients with paralytic poliomyelitis; this fact indicates that these viruses have higher neurovirulence in human beings infected naturally. In this study, the neurovirulence of these isolates was evaluated in the PVR-Tg21 transgenic mice expressing the human poliovirus receptor gene^34^,^35^. Strains CHN8184 and CHN8229-1 showed neurovirulence (PD_50_ = 2.7 and 2.8 CCID_50_ per mouse, respectively) that was comparable to that of the reference wild type 1 Mahoney strain (PD_50_ < 2.0 CCID_50_ per mouse), the cVDPV strains isolated from Hispaniola (strain HAI00-003; AF405669) and The Philippines (strain Mindanao01-1; AB180070) (PD_50_ = 2.8 and 2.4 CCID_50_ per mouse, respectively)^8^,^9^ (Table 1). In contrast, strain CHN8225c, which was isolated from a contact of the second patient in Yaoshang village (CHN8184), exhibited moderate attenuation of the neurovirulence in the PVR-Tg21 mice (PD_50_ = 4.2 CCID_50_ per mouse) (Fig. 4).

### Potential new candidate determinants of attenuation

**Figure 6:**
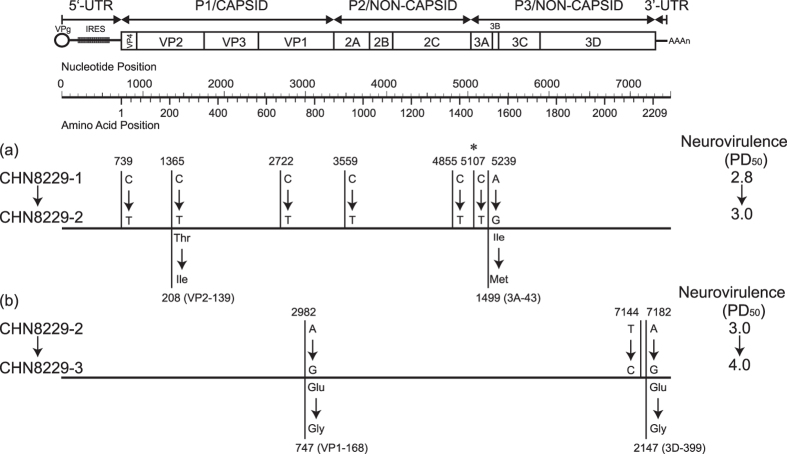
Nucleotide (upper bars) and amino acid (lower bars) substitutions into the genome of Guizou/China cVDPV strains. CHN8229-1 (**a**) and CHN8229-2 (**b**) were used as the reference sequences, respectively. Nucleotides that had reverted back to the Mahoney strain are indicated by asterisks. The change in neurovirulence is indicated on the right side of each bar.

Strains CHN8229-1, CHN8229-2, and CHN8229-3 were isolated from the successive stool specimens collected from the third patient in the outbreak, and they exhibited the trend of decreasing neurovirulence (PD_50_ = 2.8, 3.0, and 4.0 CCID_50_, respectively) along with evolution of the virus. However, there was a slight difference among the sequences of their *VP1*-coding region: a nucleotide substitution at nt2722 (C-to-T) was noted in the *VP1* region of the strain CHN8229-2 when compared with the strain CHN8229-1, and another nucleotide substitution at nt2982 (A-to-G) was noted in the *VP1* region of the strain CHN8229-3 when compared with the strain CHN8229-2. Further, in the complete genome sequences, 7 nucleotide substitutions were noted of the strain CHN8229-2 when compared with the strain CHN8229-1, which led to 2 amino acid substitutions, among them, nt5107 is another general accepted attenuating sites that had reverted back to the Mahoney strain. While only 3 nucleotide substitutions (nt2982 [A-to-G, a missense mutation], nt7144 [T-to-C, a synonymous mutation], and nt7182 [A-to-G, a missense mutation]) of the strain CHN8229-3 when compared with the strain CHN8229-2, which also led to 2 amino acid substitutions (VP1–168: Glu-to-Gly and 3D–399: Glu-to-Gly) (Fig. 6).

As it is well known, a small number of nucleotide or amino acid substitutions (or even a single nucleotide or amino acid substitution) can lead to a substantial change in the poliovirus neurovirulence^27^,^36^, based on the fact that strains CHN8229-2 (PD_50_ = 3.0) and CHN8229-3 (PD_50_ = 4.0) are ten times stronger neurovirulent, we assume that some unknown determinants of attenuation occur among these substitutions. Three nucleotide substitutions—at positions nt2982, nt7144, and nt7182—are most likely to be associated with neurovirulence; hence, a thorough research is needed to unveil the exact mechanism underlying these nucleotide substitutions for the attenuation of type 1 polioviruses.

## Discussion

A cluster of cases of poliomyelitis due to cVDPVs has been identified in an area with low OPV coverage in Guizhou Province, China^15^. The extent of accumulated nucleotide changes suggests that the first OPV dose was administered toward on April of 2003, and viral circulation continued throughout the winter. Although annual province-wide NIDs have been conducted in Guizhou Province since 1996, the data indicate that OPV coverage in some areas is declining. Poliomyelitis caused by VDPVs is now the biggest challenge for maintaining the “polio free” status in China.

Eight type 1 Guizhou/China VDPVs, belonged to two lineages (Lineage 1 and Lineage 2), as determined by sequence similarity analysis. Lineage 1 was called the strong neurovirulence lineage because it included isolates that show higher neurovirulence and caused paralysis in patients, while lineage 2 was called the weak neurovirulence lineage because it included isolates that show lower neurovirulence and did not cause paralysis (Fig. 1). Although there is some coincidence, the clinical manifestations of the patients were consistent with the animal experimental results of the cVDPV strains in this study. The data also indicate that a single cVDPV progenitor could circulate and evolve into both a highly neurovirulent lineage 1 and a less neurovirulent lineage 2; It indicates that the evolution of the polioviruses in nature is not directional; however, the mutation of some key sites may affect the neurovirulence of polioviruses during their evolution. In addition, in a same individual, a fully neurovirulent cVDPV (CHN8229-1) may involve into a less neurovirulent form (CHN8229-3), which indicates that increasing of the mutation sites in the viral genomes was not associated with higher neurovirulence, what really works is some key sites.

Guizhou/China cVDPV isolates exhibited some biological properties such as high neurovirulence and “non-Sabin-like” antigenic properties that were similar to those of wild-type polioviruses and the cVDPV isolates reported previously in Egypt, Hispaniola, the Philippines, and Madagascar^8^,^9^,^10^,^12^; however, they differed in some key aspects. Guizhou/China cVDPV isolates showed high neurovirulence even without recombination and without base pair 480-A and 525-U reversion to the Mahoney type in their genomes. And the other cVDPV isolates were highly neurovirulent with recombination with other enteroviruses species C in the noncapsid region and a base pair 480-A and 525-U reversion to the Mahoney type, which were believed to be associated with the occurrence of cVDPV-associated poliomyelitis outbreaks^6^,^7^,^8^,^9^.

Base pairing of nucleotides 480 and 525 in the 5′-UTR of the poliovirus genome in the secondary structure in IRES was considered most important for determining the neurovirulence of type 1 polioviruses^37^,^38^. Nucleotides 480-G and 525-U in the Sabin 1 strain appeared to be associated with a decrease in the neurovirulence and translation efficiency of the viral lifecycle. If there is a transition from G to A at position 480, with/without a transition from U to C at position 525, then the isolate will lack the genetic and phenotypic properties of the vaccine strain, will function as the wild-type Mahoney strain, and show higher neurovirulence and translation efficiency^39^.

It should be noted that all 8 Guizhou/China cVDPV strains contain a mutation at position 476 in the 5′-UTR region. This mutation is also located in the long stem region of domain V of the IRES and changes an unpaired U-U mismatch to an A-U base pair between nucleotides 476 and 529 thus also resulting in the strengthening of the secondary structure of domain V of the IRES and most likely in the reversion of the attenuation effect of mutation at 480 in Sabin 1. This mode of reversion was first identified in Sabin 1 viruses following serial passage in the human intestinal tract, which containing the same mutation at nucleotide 476 showed increased neurovirulence in monkeys^40^. And then this mode of reversion was also identified in viruses excreted by vaccinated children in a clinical study in the UK and revealed that mutation at nucleotide 476 accounted for 10% of all Sabin 1 revertants^41^. The mutation at 476 was also recently found in sequential isolates from an immunodeficient patient. A virus isolate containing mutations at nucleotide 476 and amino acid VP1-106 (also known to have an effect on attenuation of Sabin 1), similar to Guizhou/China cVDPV strains in this paper, showed a neurovirulent phenotype in transgenic mice comparable to that of the wild-type Mahoney strain^42^.

The conclusion is that the relevance of the mutation at nucleotide 480 for the attenuation of Sabin 1 remains the same, but reversion of the attenuation phenotype due to this mutation can occur by direct reversion at nucleotide 480, by mutation at nucleotide 525 which strengthens the base pair between nucleotides 480 and 525 or by mutation at nucleotide 476 which strengthens the base pair between nucleotides 476 and 529. The results shown here further confirm the role of the mutation at nucleotide 476 as one of the possible mechanisms for reversion as previously established in isolates from healthy individuals and strains from immunodeficient long term excretors^41^,^42^.

Three VDPV strains, CHN8229-1, CHN8229-2, and CHN8229-3, were isolated from 3 successive (1^st^, 2^nd^, and 3^rd^) stool specimens collected from the third polio patient mentioned above, but their nucleotide sequences were not identical. The results of the neurovirulence test for these 3 successively collected isolates showed that the neurovirulence decreased with the prolongation of sampling and that the PD_50_ value changed from 2.8 to 4.0, raising the possibility that reversion of the attenuated phenotypes of Sabin 1 strain is not necessarily irreversible during cVDPV evolution. Since there was little difference in the *VP1* region and whole genome sequences between the isolates, a nucleotide substitution at nt2982 in the *VP1* region (which differed between CHN8229-2 and CHN8229-3 strains) may be the most likely candidate determinant of attenuation, because the 3 strains belonging to the weak neurovirulence lineage also had nt2982-G identical to strain CHN8229-3. Moreover, nucleotide substitutions at nt7144 and nt7182 in the *3D* region also should be studied in order to determine their role in neurovirulence determination.

The Guizhou/China cVDPV strains differed in another key aspect from the isolates obtained during the outbreaks reported in Egypt, Hispaniola, and Madagascar: All the cVDPV strains were non-recombinant strains. The other cVDPV isolates described thus far^8^,^9^,^10^,^12^ have recombinant noncapsid sequences derived from other species C human enteroviruses. Each of the regions of the whole genome sequences of Guizhou/China cVDPV strains had the highest homology with the Sabin 1 strain, and no recombination was found with either type 2 or type 3 polioviruses or with other NPEVs. One of the important reasons for the absence of recombination in the Guizhou/China cVDPV strains might be their short-term circulation in the human community (14 months after the initiating dose of OPV). This could also be true for the gene recombination seen in the other cVDPV strains such as those isolated from the outbreaks in Egypt (114 months after the initiating dose of OPV)^10^, Hispaniola (31 months after the initiating dose of OPV)^8^, and The Philippines (32 months after the initiating dose of OPV)^9^, as these viruses circulate for a relatively longer period in the human community and, therefore, provide an opportunity for coinfection with an enterovirus species C.

It has been shown that some species C enteroviruses (especially coxsackievirus A13 and A17) that known to recombine with the vaccine polioviruses are frequently found in some countries where cVDPVs were found to recombine with enterovirus species C before, such as the Philippines^43^, Madagascar^44^,^45^, Cambodia^46^, and Nigeria^47^, while unfortunately it is unclear whether these particular viruses circulate intensively or not at all in the Guizhou populations. Recombination between two viruses depends on the co-infection of a given individual and cells with both viruses. Therefore, recombination between different types of vaccine polioviruses is possible in all individuals receiving a multivalent polio vaccine^23^, but recombination between polio vaccine strains and NPEVs depends on the presence in the infected host of viruses likely to recombine with poliovirus, such as wild polioviruses or certain species C enteroviruses. The frequency and rapidity of recombinant cVDPVs emergence in vaccinated individuals or through subsequent circulation will therefore depend on the density of the recombination partners of poliovirus in the human population. In this sense, recombination between polio vaccine strains and non-vaccine enteroviruses seems primarily to be an indicator of the presence and density of these enteroviruses in the population in addition to be an indicator of the duration of viral circulation in the human community.

Some neurovirulent type 2 and type 3 recombinant cVDPVs with enterovirus species C donor sequences also showed a limited number of nucleotide changes compared with Sabin strains, such as Madagascar type 2 and type 3 cVDPVs in 2005 (1.1%–2.7% and 1.0%–1.8% difference, respectively)^11^,^13^, which are similar to those found in this study and consistent with a similar period having elapsed since the initial dose of OPV have been reported. Among these type 2 cVDPVs, the 5′-UTR region, an essential element for tropism, pathogenicity, and circulation, has been replaced with that of enterovirus species C, and they were all neurovirulent in mice^13^. The study proved that VDPVs may become pathogenic in complex viral ecosystems, through frequent recombination events and mutations. Although type 1 Guizhou/China cVDPVs emerge as pathogenic viruses through mutations in the absence of recombination with NPEVs, this does not exclude the possibility that, in addition to mutations, recombination with NPEV may contribute to genetic and phenotypic changes in wild type and attenuated strains of polioviruses^48^.

The cVDPVs outbreak in Guizhou of China has important implications in the global initiative to eradicate polio: high quality surveillance permitted very early detection and response, and it played a key role in stalling the widespread circulation of the emergent cVDPV strains in China. The apparently prolonged local circulation of a VDPV lineage in a small susceptible population described in this report is important information that would enhance the limited global knowledge on the early emergence of cVDPVs. Further, the finding of highly neurovirulent polioviruses with nucleotide 480-G and without recombination indicates that some unknown nucleotide changes may substantially affect poliovirus neurovirulence, and some candidate determinants of attenuation need to be further studied to expand our knowledge about the mechanism underlying the attenuation of type 1 polioviruses.

## Materials and Methods

### Ethics Statement

This study did not involve human participants or human experimentation; the only human materials used were stool samples collected from AFP patients and health children at the instigation of the Ministry of Health P. R. of China for public health purposes, and written informed consent for the use of their clinical samples was obtained from their parents of the child patients on their behalf. This study was approved by the second session of the Ethics Review Committee of the National Institute for Viral Disease Control and Prevention, Chinese Center for Disease Control and Prevention, and the methods were carried out in accordance with the approved guidelines.

### Primary identification of the viruses

RD (human rhabdomyosarcoma cell) and L20B (murine cell line expressing the human poliovirus receptor) cell lines were used to isolate viruses from the stool specimens by using standard procedures^49^. All positive isolates were identified by a micro-neutralization test that was performed using a poliovirus-type specific rabbit polyclonal antiserum (Rijksinstituut Voor Volksgezondheid En Milieu; RIVM, The Netherlands)^49^. Poliovirus isolates were then further characterized by two ITD methods, a PCR-RFLP analysis and an ELISA method.

### Nucleic acid sequencing

Viral RNA was extracted from the poliovirus isolates by using the QIAamp Mini Viral RNA Extraction Kit (Qiagen) and was used for RT-PCR amplification by the standard method^23^. The entire *VP1* region of the poliovirus isolates was amplified by RT-PCR with primers that flanked the *VP1*-coding region by using the Access RT-PCR Kit (Promega, USA)^4^,^23^. The sequencing primers of the whole genome were designed according to the nucleotide sequence of the Sabin 1 strain. After purification of the PCR products by the QIAquick Gel Extraction Kit (Qiagen), the amplicons were bi-directionally sequenced with the ABI PRISM 3100 Genetic Analyzer (Applied Biosystems, Hitachi, Japan). The 5′ rapid amplification of cDNA ends (RACE) core set (Takara Biomedicals, Dalian, China) was used to determine the 5′ segments, according to the manufacturer’s instructions.

### Phylogenetic analysis and RNA secondary structure prediction

Sequence data were stored as standard chromatogram format (.ab1) files and analyzed using Sequencher software (version 4.0.5) (GeneCodes, Ann Arbor, Michigan, USA). Phylogenetic dendrograms were constructed using the neighbor-joining method of the MEGA program (version 5.0) (Sudhir Kumar, Arizona State University, Arizona, USA), and the topology of the trees was determined on the basis of majority rule consensus among 1000 bootstrap replicates^50^. The sequence relationships in the 3CP of the complete ORF among the eight VDPV isolates and the ancestral Sabin 1 strain were summarized in a phylogenetic tree constructed by Bayesian MCMC analysis using the BEAST program (version 1.4)^51^. The tree was rooted to the ORF of Sabin 1 strain, and the time of the initiating OPV dose and divergence of different VDPV branches was estimated from the rate of 3CP substitutions into the ORF. The ratio of nonsynonymous to synonymous substitutions (*Ka*/*Ks* ratio) within the Nag sites were determined using the Pamilo-Bianchi-Li (PBL) method implemented in the MEGA program (version 5.0)^50^. The secondary structures of domain V of the internal ribosome entry site (IRES) in the 5′-UTR region of polioviruses as previously described^52^ were folded with RNA structure software (version 5.2)^53^.

### Neurovirulence testing in PVR-Tg21 mice

A neurovirulence test was carried out using PVR-Tg21 mice that expressed the human poliovirus receptor (CD155)^34^,^35^. Type 1 reference Sabin attenuated strain (obtained from the National Institute for Biological Standard and Control [NIBSC], UK) and type 1 reference Mahoney neurovirulent strain (obtained from the National Institute of Infectious Diseases [NIID], Japan) were used as virus controls in the test. In brief, 6 mice (equal number of males and females) were assigned to 1 group and were inoculated intracerebrally with 30 μl of each virus dilution (in 10-fold increments; range: 2.5–6.5 log 50% cell culture infective dose [CCID_50_] per mouse). The mice were examined daily for 14 days after the inoculation, and the number of paralyzed or dead mice was recorded. The virus titer that induced paralysis or death in 50% of the inoculated mice (PD_50_) was calculated by using the Kärber formula and expressed as PD_50_/mouse.

### Nucleotide sequence accession numbers

Complete genome sequences of 8 type 1 Guizhou/China cVDPV strains described in this study have been deposited in the GenBank database under the accession numbers FJ769378 –FJ769385.

## Additional Information

**How to cite this article**: Zhang, Y. *et al.* An Insight into Recombination with Enterovirus Species C and Nucleotide G-480 Reversion from the Viewpoint of Neurovirulence of Vaccine-Derived Polioviruses. *Sci. Rep.* **5**, 17291; doi: 10.1038/srep17291 (2015).

## Figures and Tables

**Table 1:** Genetic and phenotypic characterization of Guizhou/China type 1 cVDPVs.

Virus	Nucleotide and amino acid of neurovirulence determinants												Recombination with EV-C	Neurovirulence (Log PD_50_)
	5′*-*UTR	5′*-*UTR	5′*-*UTR	*VP4*		*VP3*		*VP1*		*3D*		3′*-*UTR		
	nt476	nt480	nt525	nt935	aa65	nt2438	aa225	nt2795	aa106^a^	nt6203	aa73	>nt7441		
P1/Sabin	U	G	U	U	Ser	A	Met	A	Thr	C	His	G	—	>7.8
CHN8184	A	G	U	U	Ser	U	Leu	G	Ala	U	Tyr	A	No	2.7
CHN8229-1	A	G	U	G	Ala	U	Leu	G	Ala	U	Tyr	A	No	2.8
CHN8229-2	A	G	U	G	Ala	U	Leu	G	Ala	U	Tyr	A	No	3
CHN8229-3	A	G	U	G	Ala	U	Leu	G	Ala	U	Tyr	A	No	4
CHN8225c	A	G	U	U	Ser	U	Leu	G	Ala	U	Tyr	A	No	4.2
P1/Mahoney	U	A	U	G	Ala	U	Leu	G	Ala	U	Tyr	A	—	<2.0
HAI00-003^b^	U	A	U	G	Ala	A	Met	G	Ala	U	Tyr	A	Yes	2.8
Mindanao01-1^b^	U	A	U	G	Ala	A	Met	G	Ala	U	Tyr	A	Yes	2.4

The nucleotides and amino acids that shared identity with those of the Sabin 1 strain are indicated by shaded boxes.

^a^surface residues forming a part of NAg1.

^b^Representative type 1 cVDPV strains from Hispaniola and The Philippines. The PD50 value is cited from the previous reports 8,9.
